# Predictive Factors for Encephalitis Caused by *Angiostrongylus cantonensis* Infection

**DOI:** 10.4269/ajtmh.2010.09-0646a

**Published:** 2010-03

Dear Sir:

An interesting study about clinical predictive factors of encephalitis caused by *Angiostrongylus cantonensis* infection was recently published in the *American Journal of Tropical Medicine and Hygiene*.^1^ We concur with the authors' observation that age, fever, and prolonged headache are risk factors for developing encephalitic angiostrongyliasis (EA). We would extend this observation and suggest that children have been considered to have an increased mortality rate compared with adults in Taiwan's study.^2^ Although recently there have been several outbreaks among adults,^3^,^4^ particularly Thai laborers who have eaten golden apple snails (*A. canaliculatus*), most cases of *A. cantonensis* eosinophilic meningitis in Taiwan have been reported in children exposed to the African giant snail (*A. fulica*).^2^ There is usually a history of eating or playing with snails or slugs. The disease is much more serious in children. In a series of 87 cases,^5^ about 50% had fever and 30% had hepatomegaly. There were 4 deaths, and 6 patients had permanent neurologic sequelae. In the study of Sawanyawisuth and others,^1^ they enrolled only adult patients in the encephalitis group. The median age in the meningitis group was 33.5 (range 15–70), predominantly in the adult populations. Those would account for the difference in the mortality rate in terms of age between Taiwan and the Thailand study.

Brain magnetic resonance imaging (MRI) abnormalities in eosinophilic meningitis caused by *A. cantonensis* infection is relatively common. Multiple enhancing nodules in the brain and linear enhancement in the leptomeninges, accompied by stick-shaped enhancement, were the characteristic signs of the disease on Gd-DTPA-enhanced T1-weighted images.^6^ We also found that there was a significant correlation between severity of headache, cerebrospinal fluid (CSF) pleocytosis, and CSF and blood eosinophilia with MRI signal intensity in T1-weighted imaging (*P* < 0.05).^7^ However, in the study of Sawanyawisuth and others,^1^ they excluded those patients with abnormal brain computed tomography or magnetic resonance findings to aim for eliminating other possible causes of CSF eosinophilia; this would bias the patient's selection, because the more severe cases with abnormal imaging findings will be excluded by the present criteria. Finally, the samples size is not accurate. It was assumed that the assumption of numbers of patients with fever or neck stiffness in the meningitis and encephalitis groups were 10% and 40%. Using a two-sided significance level of 0.05, power of 80%, and the meningitis/encephalitis sample size ratio of 6:1, the approximate numbers of the encephalitis and meningitis groups were 19 and 116 subjects, respectively,^8^ instead of 14 and 86 described in the Sawanyawisuth study. The small number of patients in the encephalitis group will further dampen the primary limitation of the study.

These observations suggest that there is a significant difference in the clinical manifestations between patients with eosinophilic meningitis caused by *A. cantonensis* infection. Further research is also required to determine the risk factor to induce EA in *A. cantonensis* infection.

Hung-Chin Tsai

Cheng-Len Sy

Shue-Ren Wann

Susan Shin-Jung Lee

Yao-Shen Chen

Section of Infectious Diseases

Department of Medicine

Kaohsiung Veterans General Hospital

Kaohsiung, Taiwan; andNational Yang-Ming UniversityTaipei, Taiwan, R.O.C.

E-mails: hctsai1011@yahoo.com.tw

lenlensy@gmail.com

srwann@vghks.gov.tw

ssjlee28@yahoo.com.tw

vschen@vghks.gov.tw

Chuan-Min Yen

Department of Parasitology and Graduate Institute of Medicine

Kaohsiung Medical University Kaohsiung

Taiwan, R.O.C.

E-mail: chmiye@cc.kmu.edu.tw
